# Apparent diffusion coefficient as a prognostic factor in clival chordoma

**DOI:** 10.1038/s41598-020-79894-8

**Published:** 2021-01-12

**Authors:** Hyeong-Cheol Oh, Chang-Ki Hong, Kyu-Sung Lee, Yoon Jin Cha, Sung Jun Ahn, Sang Hyun Suh, Hun Ho Park

**Affiliations:** 1 Department of Neurosurgery, Gangnam Severance Hospital, College of Medicine, Yonsei University, 211 Eonju-ro, Gangnam-gu, Seoul, 06273 Republic of Korea; 2 Department of Pathology, Gangnam Severance Hospital, College of Medicine, Yonsei University, Seoul, Republic of Korea; 3 Department of Radiology, Gangnam Severance Hospital, College of Medicine, Yonsei University, Seoul, Republic of Korea

**Keywords:** Bone cancer, Bone cancer

## Abstract

Clival chordoma is a rare disease with high recurrence rates even after a combination of surgical resection and radiotherapy. Apparent diffusion coefficient (ADC) has been used to evaluate aggressive features of chordoma, but its utility for clival chordoma has not been explored specifically. In this study, the utility of preoperative ADC values was analyzed for predicting tumor progression and recurrence in patients with clival chordoma. Between 2012 and 2019, a total of 30 operated cases were analyzed with available preoperative ADC data. Receiver operating characteristic (ROC) analysis was used to obtain ADC cutoff values for predicting tumor aggressiveness. The mean and minimum ADC values were significantly lower in the aggressive tumor group than in the stable tumor group (both *P* < 0.001). ROC analysis showed that a mean cutoff ADC value of 1198 × 10^−6^ mm^2^/s and minimum ADC value of 895.5 × 10^–6^ mm^2^/s could be used to predict aggressive features of clival chordoma. Subtotal resection, partial resection, and mean and minimum ADC values that were lower than cutoff values were negative predictors of overall survival and progression-free survival. In conclusion, mean and minimum ADC values could be useful in predicting aggressiveness of clival chordoma.

## Introduction

Clival chordoma is a rare and slow-growing disease that infiltrates the bone and extends into the adjacent soft tissue, especially to the brainstem^1^. Such chordomas show malignant behavior with high recurrence rates even after treatment with the combination of maximal safe surgical resection and adjuvant radiotherapy^2^. Furthermore, adjacent neurovascular structures make it difficult to eradicate the tumor surgically^3,4^. To overcome these locally invasive properties of chordoma, several studies have examined the efficacy of chemotherapeutic agents for treating chordoma, but none were successful for clinical use^5–8^. Brachyury, a targeting transcription factor is one example that has shown to be effective, but clinical application is still limited^6^. Particle beam radiation (proton or carbon ion based) has been introduced into chordoma treatment, but its efficacy over conventional radiation remains controversial^9–12^.

Many studies have investigated prognostic factors of clival chordoma, but the understanding of the disease is still limited due to its rarity and cancer biology. Previous studies have revealed that tumor size, location, and extent of resection (EOR) are associated with overall survival (OS) and progression-free survival (PFS) in clival chordoma^4,13,14^. However, there are still no reliable factors that could predict the behavior of the tumor before treatment begins.

A recent study revealed that apparent diffusion coefficient (ADC) values could be used to predict tumor progression for craniospinal axis chordomas^15^. The study, however, covered both clival and sacral chordomas, included only a small number of patients, and did not take EOR into consideration, which is still, the most reliable prognostic factor for clival chordoma. In this study, we evaluated the aggressiveness of clival chordomas using preoperative ADC values^16^ and determined ADC cutoff values to predict tumor progression and recurrence.

## Results

### Clinical characteristics of chordoma patients

There were 16 male (53.3%) and 14 female (46.7%) patients with a mean age of 48.9 years (range 24–76 years). Histologically, two cases were diagnosed as chondroid chordoma and 28 cases as classic chordoma. Gross total resection (GTR) was achieved in 12 cases (40%), near-total resection (NTR) in 10 cases (33.3%), subtotal resection (STR) in 6 cases (20%), and partial resection (PR) in 2 cases (6.7%). Seventeen cases (56.7%) underwent adjuvant intensity-modulated radiotherapy and 13 cases (43.3%) underwent proton-beam radiotherapy.

**Figure 1:**
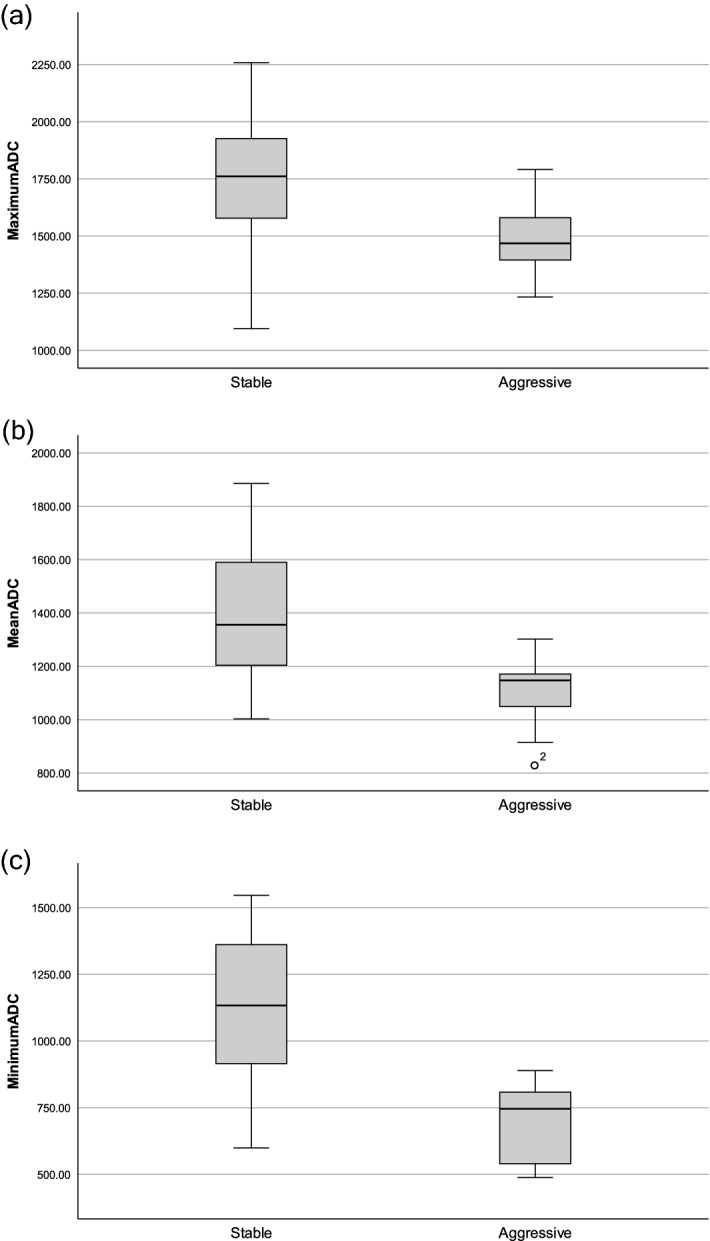
Comparison of ADC values between stable and aggressive chordoma (10^–6^ mm^2^/s). There were significant differences between groups in (**a**) maximum ADC (*P* = 0.012), (**b**) mean ADC (*P* < 0.001), and (**c**) minimum ADC (*P* < 0.001).

There were 21 cases in the stable group (70%) and 9 cases (30%) in the aggressive group. Patient and tumor characteristics are summarized in Table 1. The mean (*P* < 0.001), maximum (*P* = 0.012), and minimum (*P* < 0.001) ADC values were significantly lower in the aggressive than in the stable tumor group (Fig. 1). The interclass correlation coefficient for interobserver reliability of mean ADC values was 0.925 (95% CI, 0.843–0.967). The rates of GTR and NTR were significantly higher in the stable than in the aggressive group (*P* = 0.003). The mean number of surgical resections was higher in the aggressive than in the stable group (*P* = 0.002). There were no differences in sex, age, histology, and the type of adjuvant radiotherapy received between the two groups.

**Table 1:** Case demographics and clinical characteristics.

	Aggressive group (N = 9)	Stable group (N = 21)	*P*-value
Age at first operation (year)	42.11 ± 14.54	51.81 ± 13.37	0.087
Male	6 (66.7%)	10 (47.6%)	0.440
Pathology (Classic chordoma)	8 (88.9%)	20 (95.2%)	0.517
Extent of tumor removal (GTR + NTR)	3 (33.3%)	19 (90.5%)	0.003
Radiotherapy (Proton)	4 (44.4%)	9 (42.9%)	1
Brainstem extension (at first MRI)	9 (100%)	15 (71.4%)	0.141
Average operation number (number/patient)	2.67 ± 1.32	1.19 ± 0.40	0.002
Mean ADC (10^−6^ mm^2^/s)	1091.78 ± 147.59	1411.24 ± 256.74	< 0.001
Maximum ADC (10^−6^ mm^2^/s)	1485.44 ± 190.06	1757.90 ± 274.14	0.012
Minimum ADC (10^−6^ mm^2^/s)	702.11 ± 144.95	1125.43 ± 301.78	< 0.001

*ADC* apparent diffusion coefficient, *GTR* gross total resection, *MRI* magnetic resonance imaging, *NTR* near total resection, *SD* standard deviation. Variables are presented as mean ± standard deviation or number (percent).

### Preoperative ADC values for predicting aggressiveness of clival chordoma

**Figure 2:**
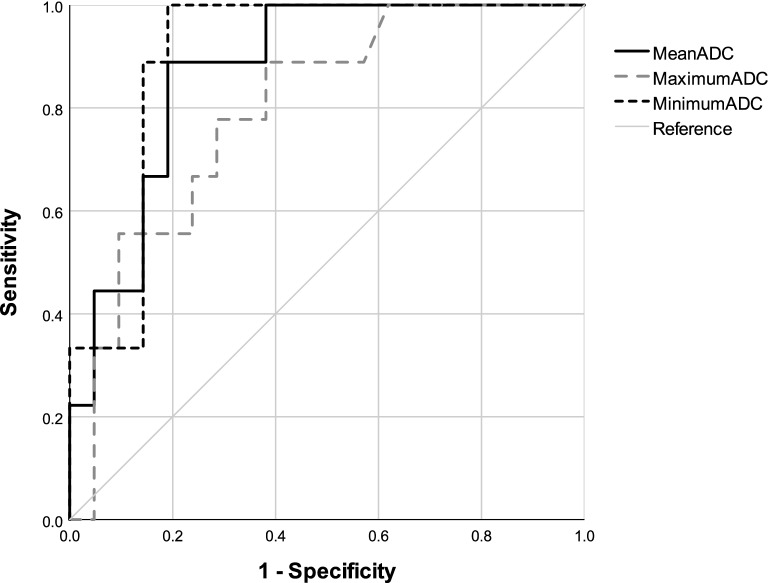
ROC curves for ADC values differentiating aggressive chordoma from stable chordoma.

According to ROC analysis, the ADC cutoff values that distinguished the aggressive from the stable tumor group were as follows: mean 1198 × 10^–6^ mm^2^/s, sensitivity of 0.89, specificity of 0.81, area under the curve of 0.873 (*P* = 0.001; 95% CI, 0.748–0.998), maximum 1695 × 10^–6^ mm^2^/s, sensitivity of 0.89, specificity of 0.62, and area under the curve of 0.796 (*P* = 0.011; 95% CI, 0.631–0.962), and minimum 895.5 × 10^–6^ mm^2^/s, sensitivity of 1, specificity of 0.81, and area under the curve of 0.899 (*P* = 0.001; 95% CI, 0.787–1.000) (Fig. 2).

### Preoperative ADC values for predicting PFS and OS of clival chordoma

**Figure 3:**
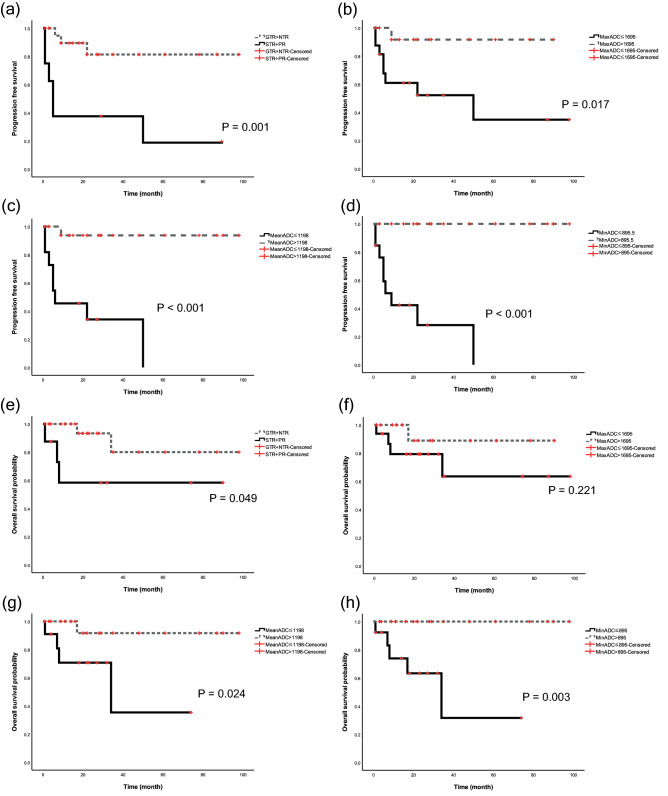
Kaplan–Meier curves using log-rank tests for overall survival and progression-free survival: (**a**) PFS according to extent of tumor resection; (**b**) PFS according to maximum ADC values; (**c**) PFS according to mean ADC values; (**d**) PFS according to minimum ADC values; (**e**) OS according to extent of tumor resection; (**f**) OS according to maximum ADC values; (**g**) OS according to mean ADC values; and (**h**) OS according to minimum ADC values.

Four patients died during a mean follow-up of 30 months (range, 1–98 months). The predictive factors associated with PFS of clival chordoma are shown in Table 2. Log-rank tests revealed that a mean ADC value ≤ 1198 × 10^–6^ mm^2^/s (*P* < 0.001), a minimum ADC value ≤ 716.5 × 10^–6^ mm^2^/s (*P* < 0.001), and maximum value ADC ≤ 1695 × 10^–6^ mm^2^/s (*P* = 0.017) correlated with a poor PFS (Fig. 3). GTR and NTR were associated with an improved PFS (*P* = 0.001). Other variables such as brain stem extension, MRI enhancement pattern, and the type of adjuvant radiotherapy received had no significance on PFS. Kaplan–Meier survival analysis showed that EOR (STR and PR) (*P* = 0.049), mean ADC value ≤ 1198 × 10^–6^ mm^2^/s (*P* = 0.024), and minimum ADC value ≤ 716.5 × 10^–6^ mm^2^/s (*P* = 0.003) were significantly associated with a poor OS. A lower maximum ADC value had a trend toward a shorter OS (*P* = 0.062).

**Table 2:** Univariable analysis of factors associated with PFS using Kaplan–Meier method.

Explanatory variables	Total No	No. of events	*P*-value
Age at first operation (year)			0.510
Younger than 50	18	7	
50 or older	12	2	
Histopathology			0.300
Chondroid	2	1	
Classic	28	8	
Adjuvant radiation therapy			0.701
Proton	13	4	
Photon	17	5	
MRI enhancement			0.136
Heterogenous	20	8	
Little or none	10	1	
Extent of tumor removal			0.001
GTR + NTR	22	3	
STR + PR	8	6	
Mean ADC			< 0.001
> 1198 (10^−6^ mm^2^/s)	19	1	
≤ 1198 (10^−6^ mm^2^/s)	11	8	
Maximum ADC			0.017
> 1695 (10^−6^ mm^2^/s)	14	1	
≤ 1695 (10^−6^ mm^2^/s)	16	8	
Minimum ADC			< 0.001
> 895.5 (10^−6^ mm^2^/s)	17	0	
≤ 895.5 (10^−6^ mm^2^/s)	13	9	
Brainstem extension (at first MRI)			0.106
Yes	24	9	
No	6	0	

*ADC* apparent diffusion coefficient, *GTR* gross total resection, *MRI* magnetic resonance imaging, *NTR* near-total resection, *PFS* progression free survival, *PR* partial resection, *STR* subtotal resection.

## Discussion

Clival chordoma is a rare, primary bone tumor arising from notochord remnants^17,18^. It can infiltrate various areas of the skull base such as the upper, middle and lower clivus, cavernous sinus, petrous bone, and upper cervical spine with or without intradural invasion^13^. Complete surgical resection is a challenge considering the deep-seated location of the tumor and the close proximity to critical neurovascular structures. In general, a maximal safe surgical resection followed by adjuvant radiotherapy is recommended, but standardized guidelines are limited owing to the rarity of the disease^17,19,20^. So far, no chemotherapeutic agents are clinically applicable and the choice of radiation modalities remains controversial^9–11^. For better understanding of the disease, several studies have investigated prognostic factors such as EOR, histopathology, and radiation modality^2,14,21^. Unfortunately, there are no preoperative factors that can predict the tumor behavior. In the present study, we performed an analysis of 30 consecutive cases using the known prognostic factors and additionally hypothesized that preoperative ADC values could be useful in predicting the aggressiveness of clival chordomas.

ADC values have been reported to be diagnostic and prognostic for central nervous system tumors^22–24^. For example, a lower mean ADC value has been known to correlate with high cellularity and mitoses in meningioma^25^. Thus, ADC maps could be applied to predict the grade of meningioma before surgery^24,26^. Recently, Yeom et al. reported that poorly de-differentiated chordomas have a lower mean, maximum, and minimum ADC values compared to classic and chondroid chordomas^27^. A lower mean ADC value has also been reported to predict OS for craniospinal axis chordomas^15^. Our results were consistent with the findings of previous reports that preoperative ADC values could be used to infer to the characteristics of tumor components and predict the behavior of clival chordoma. Diffusion measurement correlates with intra- and extra-cellular water movement^28^. Hypercellular areas have lower ADC values from reduced water motion^29^. Microscopically, classic chordoma has a low mitotic activity and a moderate cellular component within the myxoid stroma and chondroid chordoma has large components of cartilaginous materials in matrices^30^. Although chordomas have common histopathologic characteristics, their ADC values may differ according to their mixed ratios of stroma and cells. We found that the mean ADC values ranged from 900 to 1600 (10^–6^ mm^2^/s) and minimum ADC values ranged from 550 to 1400 (10^–6^ mm^2^/s) which were consistent with previous large chordoma ADC study^16^. Because the minimum ADC values reflect high cellular components of mass and affect mean ADC values, preoperative ADC measurements could be applied to predict tumor characteristics and aggressiveness for clival chordomas.

We have found that mean, maximum, and minimum ADC values in aggressive chordoma were significantly lower than in stable cases. If there were ADC components with values lower than the cutoff values in preoperative tumor region of interests (ROIs), the tumor exhibited aggressive features even if a GTR or NTR had been achieved. If the mean and minimum ADC values in the tumor ROIs were higher than the cutoff values, the chordoma followed a stable course even with a PR. Subsequently, we could predict the prognosis of clival chordoma using preoperative ADC imaging.

In this study, cases with mean and minimum ADC values that were higher than the cutoff values showed significantly improved OS and PFS compared to those with lower values. Previous studies also correlated with our findings as cases with mean ADC values higher than 1494 (10^–6^ mm^2^/s) were significantly associated with longer OS (*P* = 0.006)^15^. In terms of PFS, the predictive value of preoperative ADC imaging is high considering the notoriously high recurrence rates of clival chordomas. Our results confirmed that preoperative ADC values lower than cutoffs could significantly predict early tumor progression and recurrence as cases with mean ADC values lower than 1198 (10^–6^ mm^2^/s) showed a median PFS of 6 months. Therefore, a closer follow-up might be ideal for cases with a lower mean ADC value.

In the current study, we analyzed the cases further, based on other well-known prognostic factors such as EOR, histology, and the type of radiotherapy received. Maximal surgical resection has shown to improve PFS and OS. Our results also demonstrated that GTR/NTR significantly prolonged PFS and OS. However, a maximal surgical resection comes at a cost with neurological deficits ranging 33–80%^3,31^.

There has been controversy that chondroid chordoma is associated with better outcomes compared to classic chordoma^32,33^. Some authors reported that the prognostic significance of histologic type is unclear^17,34,35^. Hug and Slater even revealed that non-chondroid type chordoma correlated with longer OS^35^. Although current study includes only two chondroid chordoma, our results confirmed that histologic type did not affect the prognosis. In addition, our study revealed that the same classic chordoma could have a different prognosis depending on the preoperative ADC values.

The question of which radiation modality is most effective for treating clival chordoma also remains controversial^10,11^. In general, photon-based radiotherapy has been used as adjuvant treatment following surgery. However, some reported that conventional radiotherapy might not be effective, while others suggested that there is no benefit of using proton- over photon-based radiotherapy for the treatment of clival chordomas^2^. Although our study has small sample size with relatively low statistical power, our results indicate that the radiation modality might not affect the prognosis of chordoma.

Our findings suggest that measurement of ADC can be a useful tool to predict prognosis of clivus chordoma and will help decide therapeutic plans. Regardless of ADC values, maximal safe surgical resection followed by conventional dose radiotherapy is recommended for treatment of clival chordoma. But if a chordoma belongs to the aggressive group, taking account of the higher possibility of tumor recurrence, we can consider total resection of the tumor followed by high dose radiotherapy which is higher than the conventional dose such as 50 to 66Gy^9^. Furthermore, we recommend to follow up this aggressive group patient’s imaging more closely to find the early recurrence.

There are several limitations to this study. First, the study was retrospective in nature with a small sample size. Clival chordoma is a rare disease entity to conduct a large-scale, prospective study. Second, the follow-up period was not long enough considering the slow-growing nature of chordoma. Our follow-up might not have been longer that some publications^14,21^, but our data was collected for more than 5 years. Lastly, two neurosurgeons and two neuroradiologists independently outlined the ROI with a freehand and measured ADC values. Regardless of these limitations, our results suggest that preoperative ADC values could be useful in predicting the prognosis of patients with clival chordomas. Previously, there have been a study highlighting the importance of ADC values in predicting aggressive of craniospinal axis chordomas^15^. However, sacral chordomas can have different ADC values compared to clival chordoma and higher ADC values may predict non-responsiveness to carbon ion radiotherapy^36^. Therefore, it is important to distinguish clival chordomas from other spinal axis tumors for a specific and precise analysis. In this respect, our study is the first investigation to analyze the significance of ADC values confined to clival area.

In conclusion, ADC values have been reported to be diagnostic and prognostic for central nervous system tumors. We have found that mean, maximum, and minimum ADC values in aggressive chordoma were significantly lower than in stable cases. Mean and minimum ADC values that were higher than the cutoff values showed significantly improved OS and PFS compared to those with lower values. Thus, preoperative MRI with diffusion sequence could be useful in predicting the outcome of patients with clival chordomas.

## Methods

### Study population

We conducted a retrospective single institution analysis of 32 patients who underwent surgery for clival chordomas between January 2012 and December 2019. Six patients without preoperative ADC imaging and values were excluded from the study. Of the 26 patients, a total of 30 surgical cases were performed and enrolled for the study. The cases were classified into either stable or aggressive group. The stable group was defined as having no evidence of tumor on consecutive magnetic resonance imaging (MRI) follow-ups. The aggressive tumor group was defined as evidence of tumor recurrence or progression on consecutive MRI follow-ups. ADC cutoff values for predicting the aggressiveness of clival chordoma were obtained by comparing preoperative ADC values between the two groups. The cutoff values were then analyzed for PFS and OS. Tumor recurrence was defined as a newly appearing mass on MRI at or near the surgical site. Tumor progression was defined as residual tumor size increase compared to prior MRI scan. The current study design and use of clinical data was approved by Gangnam severance hospital institutional review board (2020-0200-003). All experiments were carried out in accordance with approved guidelines. Informed consent was waived by the ethics committee (Gangnam Severance Hospital Institutional Review Board) for the entire study.

Gross total resection (GTR) was defined as complete tumor removal confirmed by the surgeon intraoperatively, and no evidence of residual tumor on 3-month and 1-year postoperative MRI scans. Near-total resection (NTR) was defined as more than 95% tumor resection with a thin layer of residual tumor on a neurovascular structure. Subtotal resection (STR) and partial resection (PR) were defined as 90–95% and less than 90% tumor resection on 3-month and 1-year postoperative MRI scans, respectively.

### Analysis of MRI data

All patients were imaged with 1.5 T MRI device (Optima MR450w GEM, GE Healthcare, Milwaukee, WI, USA). Our MRI protocol for clival chordoma included routine diffusion-weighted sequences (TR/TE, 5500/66.1 ms; slice thickness/intersection gap, 4/1 mm; matrix size, 160 × 160; FOV, 240 × 240 mm; three directions; b-value = 0 and 1000 s/mm^2^; number of averages = 2; number of slices = 35; bandwidth = 1953.12; acquisition time = 8 min 31 s), and T2-weighted fast-spin-echo sequences (repetition time/echo time (TR/TE), 5414/96 ms). After intravenous gadolinium-based contrast agent was administered at a dose of 0.1 mmol/kg body weight, axial fluid-attenuated inversion recovery sequences (TR/TE/inversion time (TI), 4000/80/2000 ms) and 3D T1 fast-spoiled gradient-recalled sequences (TR/TE, 8.2/3.2 ms; flip angle 12°; slice thickness, 1 mm; matrix size, 256 × 256; FOV, 220 × 220 mm) were taken sequentially. ADC values were automatically calculated by the operating console of the MRI device and were displayed as corresponding ADC maps.

**Figure 4:**
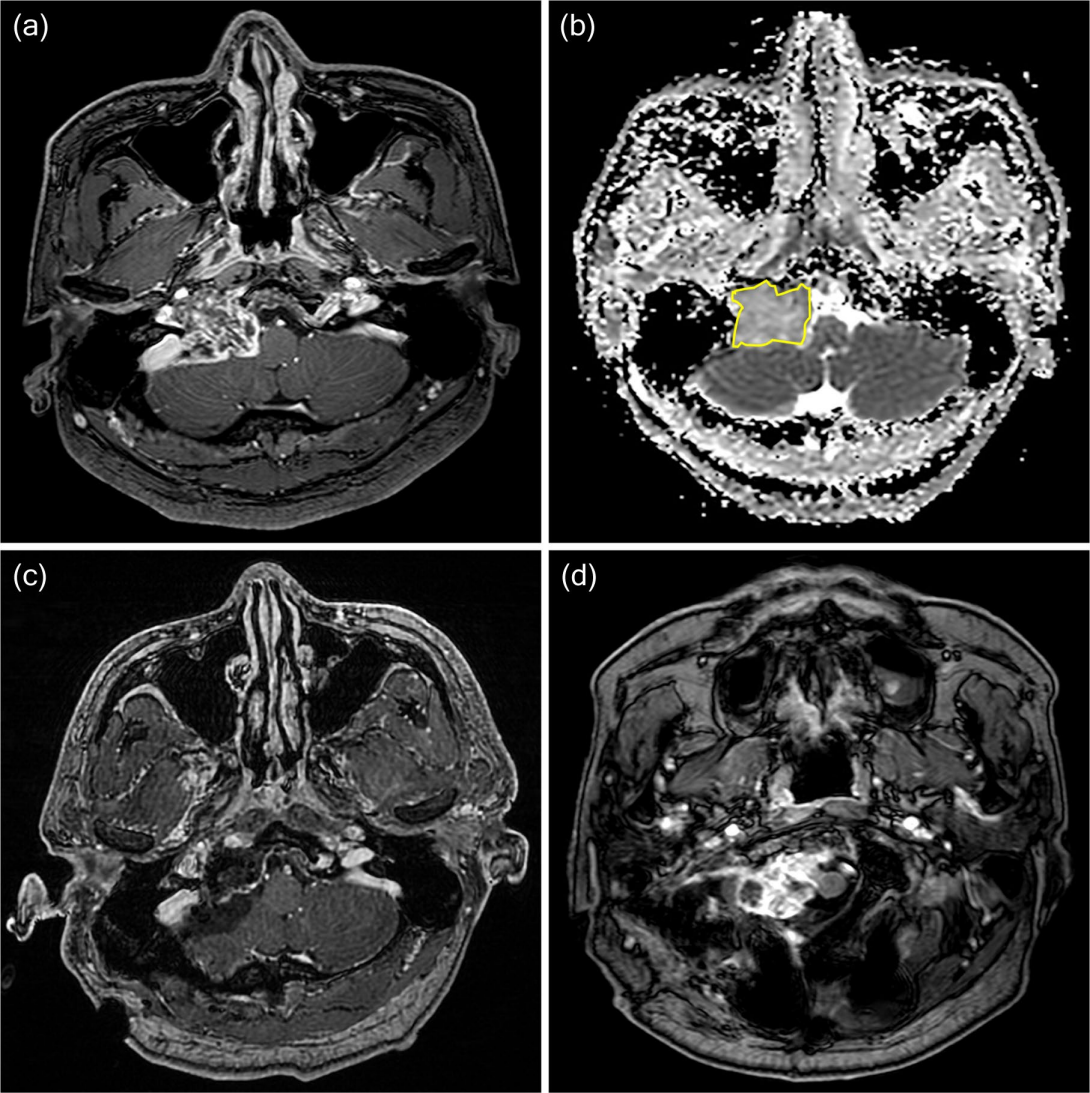
A 37-year-old man was diagnosed with classic chordoma and placed in the aggressive group. (**a**) Preoperative contrast enhanced T1-weighted imaging showed a tumor compressing the brainstem. (**b**) The ROI is outlined in yellow on the ADC map and represents decreased water diffusivity (mean ADC 1107 × 10^–6^ mm^2^/s, minimum ADC 877 × 10^–6^ mm^2^/s). (**c**) Immediate postoperative contrast enhanced T1-weighted imaging shows gross total removal. Surgery was performed via the far lateral transcondylar approach. The patient received proton-based radiotherapy 3 months after surgery. (**d**) Contrast-enhanced T1 weighted imaging obtained 2 years later showed an increase of the mass adjacent to the previous surgical site. The patient died of disease 7 months after the last MRI due to meningitis.

**Figure 5:**
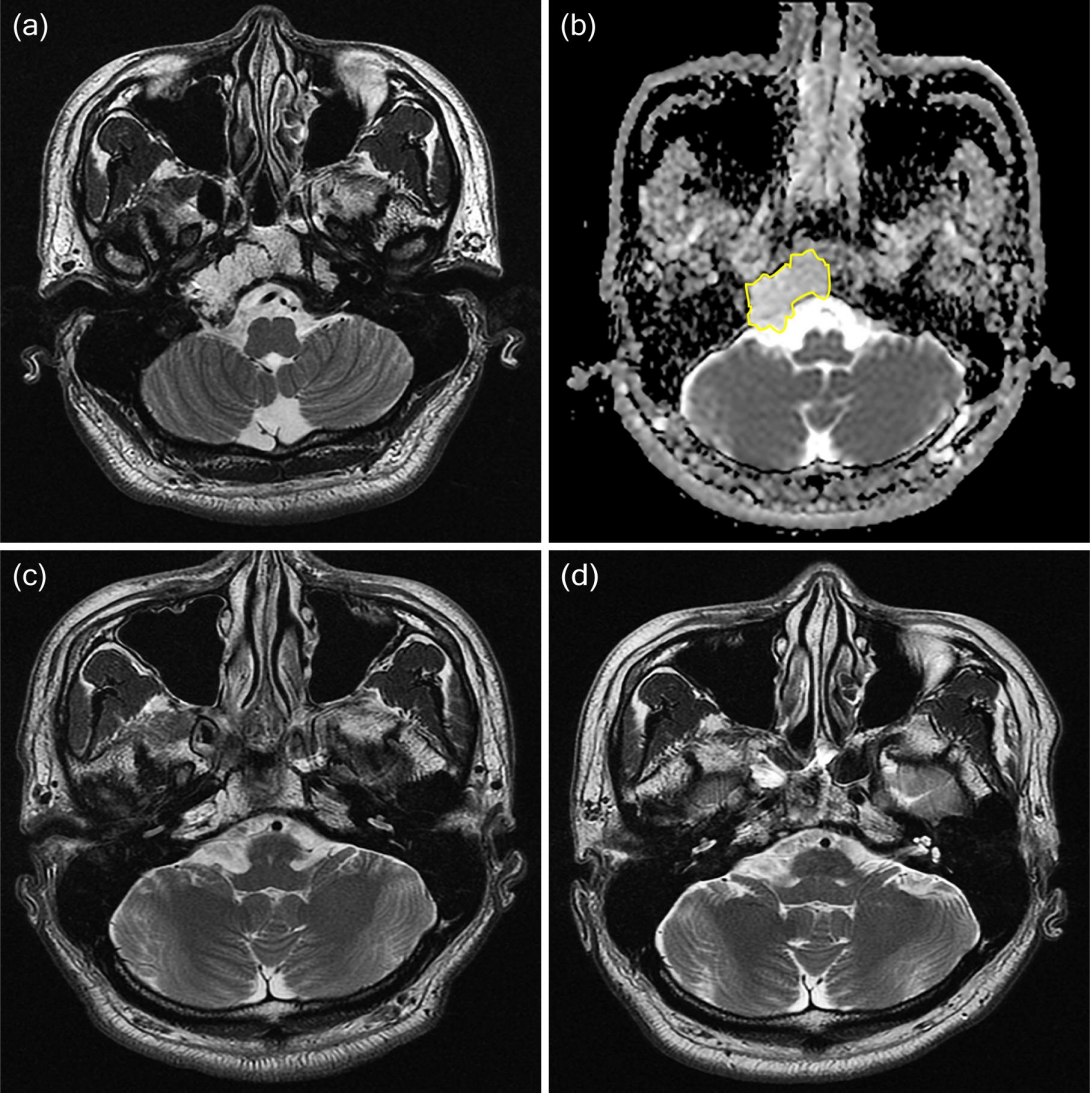
A 36-year-old man diagnosed with classic chordoma and placed in the stable group. (**a**) Preoperative T2-weighted imaging showed a T2 high signal mass arising from the clivus. (**b**) The ROI is outlined in yellow on the ADC map and represents moderate water diffusivity (mean ADC 1659 × 10^–6^ mm^2^/s, minimum ADC 1386 × 10^–6^ mm^2^/s). (**c**) Immediate postoperative T2-weighted imaging showed partial tumor removal. The operation was performed via the transsphenoidal approach. (**d**) T2-weighted follow-up imaging obtained 7 years later showed stable disease. The patient received conventional radiotherapy (62.5 Gy) 1 month after the first surgery.

Two neurosurgeons and two neuroradiologists independently outlined the region of interest (ROI) in freehand to obtain ADC values. An ROI was drawn on all axial ADC maps that included the tumor, while checking the T2-weighted and contrast enhanced T1-weighted images (Figs. 4 and 5). ADC values < 10 × 10^–6^ mm^2^/s were considered as artifacts. The mean, maximum, and minimum ADC values were obtained within this ROI for all axial sections that included the tumor and then calculated for the mean values. The ADC measurements were assessed for interobserver reliability using the interclass correlation coefficient. We also examined the enhancement pattern of each tumor and classified as heterogeneous, little, or none^27^.

### Statistical analysis

Mean, maximum and minimum ADC values, patient age at the time of first surgery, sex, EOR, histopathology, and the type of adjuvant radiotherapy received were compared between the two groups using Fisher’s exact test. The cutoff ADC values were assessed using receiver operating characteristic (ROC) analysis to predict tumor aggressiveness. PFS and OS were analyzed using Kaplan–Meier curves and compared between the groups using log-rank tests with the following variables: mean, maximum and minimum ADC cutoff values, age at the time of the first surgery, histopathology, adjuvant radiation therapy, EOR, MRI enhancement pattern, and brainstem extension. All statistical analyses were performed using IBM SPSS statistics version 25.0 (IBM Corp, Armonk, NY, USA). Two-tailed *P* values < 0.05 were considered statistically significant.
